# A Potent Tyrosinase Inhibitor, (*E*)-3-(2,4-Dihydroxyphenyl)-1-(thiophen-2-yl)prop-2-en-1-one, with Anti-Melanogenesis Properties in α-MSH and IBMX-Induced B16F10 Melanoma Cells

**DOI:** 10.3390/molecules23102725

**Published:** 2018-10-22

**Authors:** Chang Seok Kim, Sang Gyun Noh, Yujin Park, Dongwan Kang, Pusoon Chun, Hae Young Chung, Hee Jin Jung, Hyung Ryong Moon

**Affiliations:** 1 College of Pharmacy, Pusan National University, Busan 46241, Korea; autoimmuneds@naver.com (C.S.K.); rskrsk92@naver.com (S.G.N.); pyj10@pusan.ac.kr (Y.P.); 3607@pusan.ac.kr (D.K.); hyjung@pusan.ac.kr (H.Y.C.); 2 Interdisciplinary Research Program of Bioinformatics and Longevity Science, College of Pharmacy, Pusan National University, Busan 46241, Korea; 3 College of Pharmacy and Inje Institute of Pharmaceutical Sciences and Research, Inje University, Gimhae, Gyeongnam 50834, Korea; pusoon@inje.ac.kr

**Keywords:** (*E*)-3-(2,4-dihydroxyphenyl)-1-(thiophene-2-yl)prop-2-en-1-one, anti-melanogenesis, thiophene chalcone, tyrosinase inhibitor

## Abstract

In this study, we designed and synthesized eight thiophene chalcone derivatives (**1a**–**h**) as tyrosinase inhibitors and evaluated their mushroom tyrosinase inhibitory activities. Of these eight compounds, (*E*)-3-(2,4-dihydroxyphenyl)-1-(thiophen-2-yl)prop-2-en-1-one (**1c**) showed strong competitive inhibition activity against mushroom tyrosinase with IC_50_ values of 0.013 μM for tyrosine hydroxylase and 0.93 μM for dopa oxidase. In addition, we used enzyme kinetics study and docking program to further evaluate the inhibitory mechanism of **1c** toward tyrosinase. As an underlying mechanism of **1c** mediated anti-melanogenic effect, we investigated the inhibitory activity against melanin contents and cellular tyrosinase in B16F10 melanoma cells. As the results, the enzyme kinetics and docking results supports that **1c** highly interacts with tyrosinase residues in the tyrosinase active site and it can directly inhibit tyrosinase as competitive inhibitor. In addition, **1c** exhibited dose-dependent inhibitory effects in melanin contents and intracellular tyrosinase on α-MSH and IBMX-induced B16F10 cells. Overall, our results suggested that **1c** might be considered potent tyrosinase inhibitor for use in the development of therapeutic agents for diseases associated with hyperpigment disorders.

## 1. Introduction

Melanogenesis is a process that involves melanin synthesis, transport of melanin, and release of melanosome. Melanin, composed of pheomelanin and eumelanin, is synthesized in melanosomes of melanocytes [1]. The abnormal synthesis of melanin leads to skin conditions such as vitiligo, melasma, chloasma freckles, and inflammatory pigmentation. During melanins production, melanogenic enzymes such as tyrosinase was synthesized [2,3,4,5]. Tyrosinase (EC 1.14.18.1), a multifunctional copper-containing polyphenol oxidative enzyme, is key for melanin biosynthesis and is responsible for melanization in animals and browning in plants [6]. The reactions in the melanin biosynthetic pathway involves the hydroxylation of l-tyrosine to l-3,4-dihydroxyphenylalanine (l-DOPA) and the oxidation of l-DOPA to DOPA-quinone, which are important in the early stages of melanogenesis [7,8]. Thus, inhibition of tyrosinase could be a prospective target for skin pigmentation. Therefore, development of tyrosinase inhibitors for use as preservatives in a fresh food or a skin-whitening agents is under development.

Chalcones (1,3-diary-2-propen-1-ones), members of the polyphenolic and flavonoid families, exert several biological activities, including anti-bacterial [9], anti-cancer [10,11], anti-inflammatory [12], anti-tumor [13], and anti-oxidant [14] activities. Many researchers reported the inhibitory activity of chalcone-like derivatives from natural origins against mushroom tyrosinase [15,16,17]. Moreover, biological activities of chalcone-like derivatives are significantly determined based on the position and numbers of functional groups attached to benzene ring. In spite of diverse structural moiety of natural tyrosinase inhibitors and available methodology to reasonably design effective synthetic derivatives, many synthetic tyrosinase inhibitors with novel skeletons have been studied [15,16,17,18,19,20,21,22,23,24,25,26]. Especially, Hwang et al. [27] reported the effects of heterocyclic chalcone derivatives containing heterocycles, such as thiophene or furan ring as an isostere of benzene ring on free radical scavenging activity. Recently, Beak et al. [28] revealed that certain synthetic dihydroxychalcones analogues including thiophene ring inhibited cathepsin. Regardless of the evidence suggests that chalcone-like derivatives may be useful as therapeutic agents, inhibitory effect of chalcones bearing thiophene moiety on tyrosinase and melanogenesis associated with hyperpigmentation has not been reported yet. In this regard, the objective of the present work was to identify the anti-tyrosinase activity of chalcone derivatives bearing thiophene using in vitro enzyme assays with kinetics methods, including Lineweaver-Burk and Dixon plots.

Recently, our laboratory discovered and reported several tyrosinase inhibitors [18,19,20,21,22,23,24,25,26,29]. In particular, Kim et al. [22,23] and Lee et al. [24] demonstrated the importance of a thiazolidine derivative bearing 2,4-dihydroxy phenyl moiety (MHY498) which attributed to the inhibitory activity against tyrosinase. Therefore, we designed and synthesized eight compounds (**1a**–**h**) of the chalcone skeleton bearing a thiophene ring, which supported our investigation of this particular moiety. Among these synthetic compounds, a thiophene ring with 2,4-dihydroxy group on the B ring chalcone derivative (**1c**) exhibited the greatest tyrosinase inhibition and was selected for further examination. In this study, we characterize and evaluate of **1c** of its tyrosinase inhibitory activity and the modulatory role in melanin synthesis using B16F10 murine melanoma cells. The assays was performed by using a mushroom tyrosinase enzyme and melanin contents on α-MSH and IBMX-induced B16F10 melanoma cells. In addition, an inhibition enzyme kinetics study and an in silico docking program were performed in order to study inhibitory mechanism of **1c** to tyrosinase. Since there is no detailed information on the mode of inhibition and molecular interactions between tyrosinase and thiophene ring with 2,4-dihydroxy group of chalcone derivative, this study investigates an approach to develop thiophene ring chalcone derivatives as a potent anti-melanogenic drug candidate by scrutinizing molecular docking analysis and in vitro cell experiments.

## 2. Results and Discussion

### 2.1. Chemistry

**Scheme 1:**
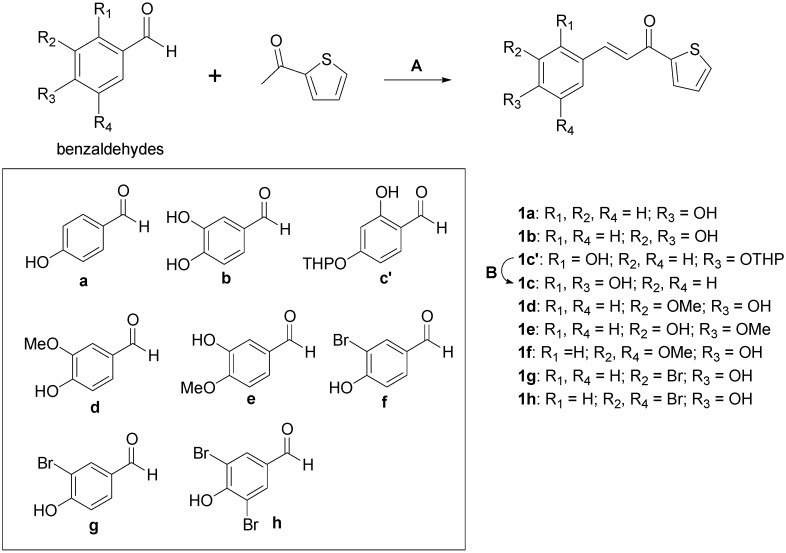
Synthetic scheme for the target compounds **1c**–**h**. Reagents and conditions: (**A**) 1.0 M HCl acetic acid solution, RT for **1a**, or 60 °C for **1b**, and **1d**, 27–96 h for **1a**, **1b** and **1d**; 1.0 M NaOH, MeOH, RT for **1e**, or 60 °C for **1f**–**h**, 1–8 h for **1e**–**h**; 20% NaOH, MeOH, RT, 48 h for **1c**′; and (**B**) 2.0 M HCl, 1,4-dioxane, RT, 30 min.

Preparation of 3-(substituted phenyl)-1-(thiophen-2-yl)prop-2-en-1-one analogs **1a**–**h** was accomplished using three synthetic methods, as depicted in Scheme 1. Compounds **1a**, **1b**, and **1c** were synthesized under acid conditions. Condensation of appropriate benzaldehydes (**a**, **b**, and **d**) with 2-acetylthiophene in the presence of a 1.0 M HCl acetic acid solution produced the desired compounds **1a**, **1b**, and **1d**. Compounds **1e**–**h** were prepared under basic conditions. Coupling of appropriate benzaldehydes (**e**, **f**, **g**, and **h**) with 2-acetylthiophene in the presence of a 1.0 M NaOH aqueous solution gave the desired compounds **1e**–**h**. However, compound **1c** was not obtained from a coupling reaction between 2,4-dihydroxybenzaldehyde and 2-acetylthiophene under either conditions. Interestingly, reaction of 2-acetylthiophene with 2-hydroxy-4-((tetrahydro-2*H*-pyran-2-yl)oxy)benzaldehyde, **c**′, instead of 2,4-dihydroxybenzaldehyde, under basic conditions produced the coupling product **1c**′, which can be hydrolyzed to give the desired compound **1c**. Treatment of **1c**′ with a 2.0 M HCl solution produced **1c** via hydrolysis of the tetrahydropyranyl (THP) group.

### 2.2. Inhibitory Effect of Compound ***1c*** on Mushroom Tyrosinase

**Figure 1:**
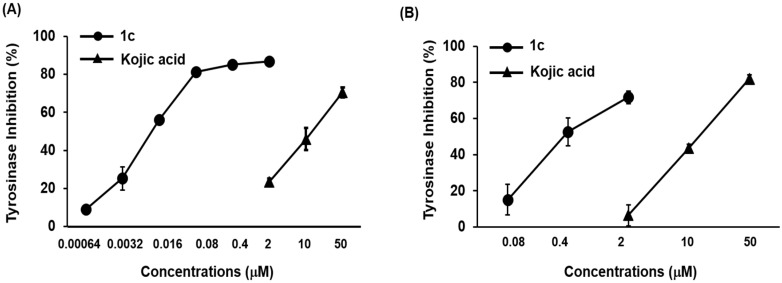
Concentration-dependent inhibitory effects of compound **1c** and kojic acid on the activity of tyrosinase for l-tyrosine (**A**) and l-DOPA (**B**) as substrates at 25 °C. The error bar indicates the standard error of the mean (SEM) of triplicate experiments.

3-(Substituted phenyl)-1-(thiophen-2-yl)prop-2-en-1-one derivatives (**1a**–**h**) were synthesized as candidate agents for skin whitening and tested for their inhibitory effects on mushroom tyrosinase. As shown in Table 1, **1c** demonstrated the most potent tyrosinase inhibitory activity, with 50% inhibition concentration (IC_50_) values of 0.013 ± 0.64 μM for monophenolase (l-tyrosine) and 0.93 ± 0.22 μM for diphenolase (l-DOPA) as substrates, respectively. For comparison, the IC_50_ values of positive control kojic acid were 22.84 ± 0.09 μM and 24.57 ± 0.23 μM. In the present study, we found that the 2,4-dihydroxy functional group on the benzylidene (**1c**) notably inhibited tyrosinase activity. This inhibitory activity was also previously shown for benzylidene-pyrrolidinedione, benzylidene-thiohydantoin, and benzylidene-thiazolidinedione derivatives as reported by our laboratory [25,26,27,28,29,30] inconsistent with in this present study. In addition, our structure–activity relationship data also demonstrated that additional hydroxyl group in the presence of a 4-hydroxy group affect tyrosinase inhibitory activity in a structurally dependent manner (**1a** vs. **1b**; **1a** vs. **1c**). Moreover, **1e** significantly inhibited tyrosinase activity, with IC_50_ values of 17.44 ± 1.81 μM and 28.72 ± 1.98 μM for both substrate l-tyrosine and l-DOPA, respectively. Recently, we reported the importance of a 3-hydroxy-4-methoxybenzylidene moiety, which contributed to improved activity toward tyrosinase [20]. Furthermore, compounds **1a**, **1b**, **1d**, and **1f** moderately inhibited tyrosinase activity toward l-tyrosine with IC_50_ values of 46.16 ± 0.55 μM, 75.72 ± 2.46 μM, 98.78 ± 2.11 μM, and 77.91 ± 8.74 μM, respectively, while **1g** and **1h** were inactive. Likewise, **1a**, **1b**, and **1g** showed moderate activity toward l-DOPA with IC_50_ values of 60.05 ± 7.85 μM, 103.44 ± 8.47 μM, and 112.09 ± 14.27 μM, respectively. However, **1d**, **1f**, and **1h** did not inhibit tyrosinase activity toward l-DOPA at any of the tested concentrations. Our results suggest that the capacity of these compounds to inhibit tyrosinase is affected by number and location of functional groups on the phenyl ring. Out of the tested compounds **1a**–**h**, **1c** was most effective in inhibiting tyrosinase activity response to l-tyrosine and l-DOPA as substrates (Table 1). Additionally, **1c** dose-dependently inhibited tyrosinase activity (Figure 1). In accordance to IC_50_ values, **1c** exhibited the highest tyrosinase inhibitory activity, and was used in consequent studies. The inhibitory effect on tyrosinase is conferred the 2,4-dihydroxyl group of benzene ring. Thus, we found that the catechol moiety (2,4-dihydroxyl groups) of **1c** plays a crucial role in tyrosinase inhibition.

### 2.3. **1c** Inhibition of Tyrosinase and Kinetic Analysis

**Figure 2:**
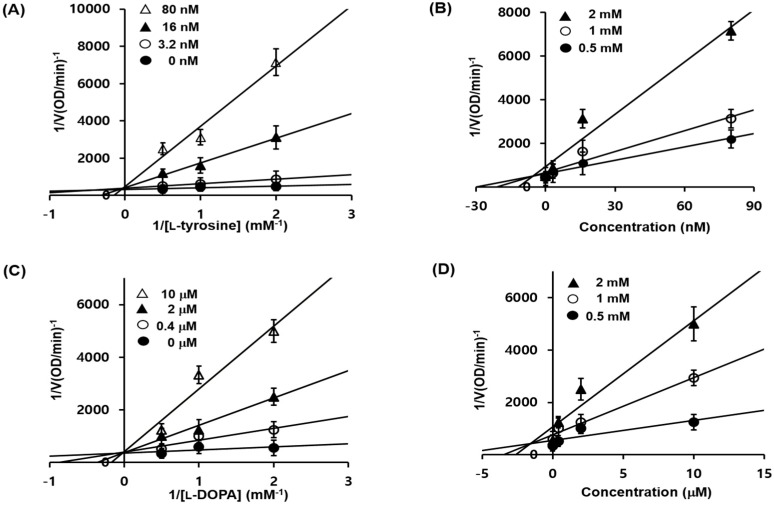
Lineweaver-Burk and Dixon plots for tyrosinase enzyme inhibition of by compound **1c**. Tyrosinase inhibition was analyzed in the presence of different sample concentrations as follows: 0 nM (filled circles), 3.2 nM (open circles), 16 nM (filled triangles), and 80nM (open triangles) (**A**). Effect of **1c** in the presence of different concentrations of substrate (l-tyrosine): 0.5 mM (filled circles), 1 mM (open circles), and 2 mM (filled triangles) (**B**). Tyrosinase inhibition was analyzed in the presence of different sample concentrations as follows: 0 μM (filled circles), 0.4 μM (open circles), 2 μM (filled triangles), and 10 μM (open triangles) (**C**). Effect of in **1c** the presence of different concentrations of substrate (l-DOPA): 0.5 mM (filled circles), 1 mM (open circles), and 2 mM (filled triangles) (**D**). The error bar indicates SEM, *n* = 3.

To determine the inhibitory mechanism of **1c**, we used two kinetic models: Lineweaver–Burk plots and Dixon plots [31,32]. Enzyme inhibition was evaluated using various concentrations of **1c** different concentrations of l-tyrosine and l-DOPA as substrates, respectively. Thus, we performed kinetic analyses using Lineweaver-Burk and Dixon plots as summarized in Table 2. According to Table 2 and Figure 2A,C, a graphical analysis of the Lineweaver-Burk plot for **1c** against tyrosinase showed competitive inhibition, as treatment with **1c** resulted in double reciprocal straight-line plots with different slopes that increased the y-axis at the same point. These data suggested that **1c** can competitively interact with substrate-binding site in order to inhibit enzyme activity when co-treated with various concentrations of l-tyrosine or/and l-DOPA as substrates. A Dixon plot is a well-accepted method for analyzing the enzyme inhibition type and determining the *K*_i_ value for an enzyme-inhibitor complex, wherein the *K*_i_ is represented by the value of the x-axis. As shown in Table 2, the *K*_i_ values were 5.01 nM and 1.76 μM, for l-tyrosine and l-DOPA substrates, respectively. The *K*_i_ values represents concentrations required to form an enzyme inhibitor complex; thus, inhibitors with lower *K*_i_ values means greater tyrosinase inhibitory activity, suggesting it could be useful in the development of preventive and therapeutic agents.

### 2.4. Molecular Docking Simulation of Tyrosinase and Binding Residues Interacting with ***1c***

**Figure 3:**
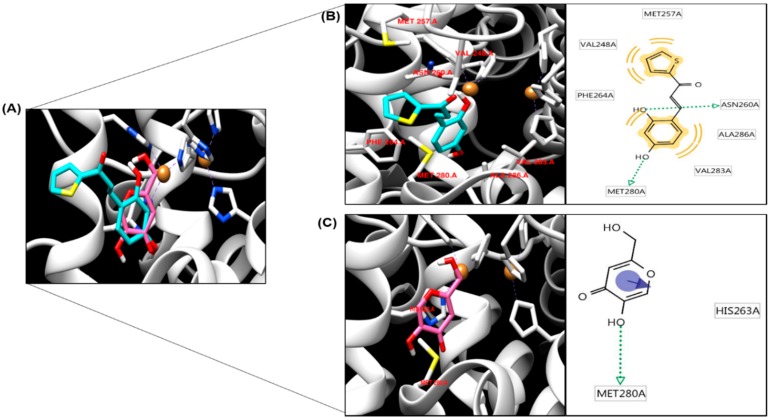
Docking simulation of compound **1c** and kojic acid with tyrosinase and pharmacophore analysis. Docking simulation results between compound **1c** and kojic acid toward mushroom tyrosinase (**A**). Pharmacophore results for **1c** (**B**) and kojic acid (**C**) obtained using LigandScout 3.1.2 showing hydrogen-bonding (green arrow), π-π stacking (violet arrow) and hydrophobic (yellow) interactions.

To understand the mechanism by which **1c** inhibits enzymatic activity of tyrosinase, we conducted in silico molecular docking simulation using AutoDock 4.2 software (SantaFe, NM, USA). Kojic acid has been widely used as a selective competitive inhibitor in numerous studies [33,34]. The docking simulation was effective with significant scores. The binding energies of **1c** and kojic acid were −5.9 and −4.21 kcal/mol, respectively. **1c** was found to exhibit greater binding affinity than kojic acid. The docking score between the ligand and the receptor could be represented by different energy source terms including electrostatic energy, Van der Waals energy, and salvation energy. Furthermore, **1c** interacted by hydrogen bonding with MET280 and ASN260 (Figure 3; Table 3). MET257, VAL248, PHE264, VAL283, and ALA286 residues were involved in hydrophobic interactions with **1c** (Figure 3B). Kojic acid was the reported compound, interacted with two active-site amino acid residues, HIS263 and MET280, with two hydrogen bonds. MET 280 participated in one hydrogen bonding interaction with the oxygen atom of kojic acid. These results proposed that **1c** is a high affinity enzyme inhibitors capable of binding to the active site of tyrosinase.

### 2.5. ***1c*** Inhibited the Melanin Content and Intracellular Tyrosinase Activity in B16F10 Melanoma Cells

**Figure 4:**
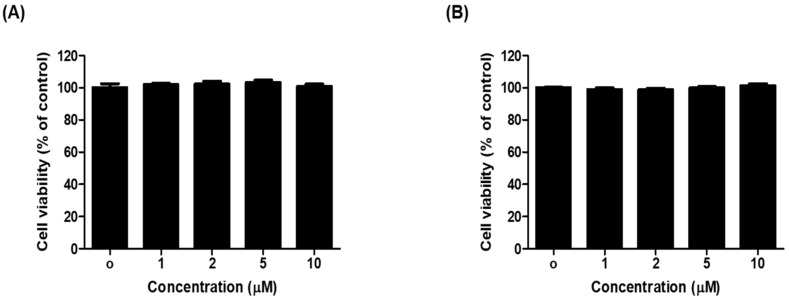
The effect of compound **1c** on cell viability of B16F10 melanoma cells. Cells were treated with various concentrations of **1c** for 24 h (**A**) and 48 h (**B**), and then examined by EZ-cytox assay. Data are representative of three separate experiments. Bars indicate standard errors of means (SEM).

**Figure 5:**
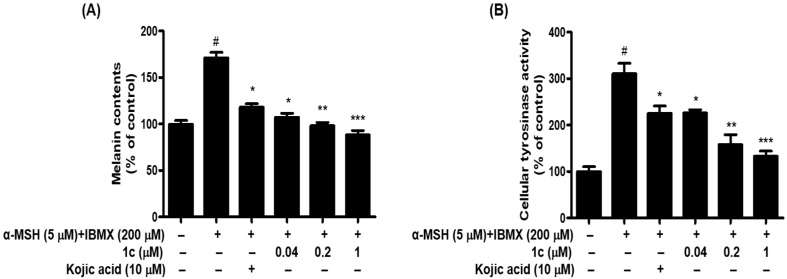
Effects of compound **1c** on melanin contents (**A**) and cellular tyrosinase activity (**B**). B16F10 cells were exposed to α-MSH (5 μM) and IBMX (200 μM)-treated with **1c** or kojic acid for 48 h. Data are representative the data of three separate experiments. Significance of **1c** or kojic acid compared with vehicle-treated control, ^#^ *p* < 0.05; significance of **1c** or kojic acid compared with α-MSH and IBMX-treated group, * *p* < 0.05, ** *p* < 0.01 and *** *p* < 0.001. Bars indicate standard errors of means (SEM).

We examined whether **1c** is cytotoxic to B16F10 melanoma cells. Treatment with **1c** did not show any cytotoxicity at concentrations up to 100 μM, as revealed by 48 h cell viability assay (Figure 4). Cytotoxic effect of **1c** was determined in B16F10 melanoma cells. The obtained result indicated no significant cytotoxicity up to 100 μM tested concentration in cells. To further test the effect of **1c** on anti-melanogenesis, cells were treated with 0.04, 0.2, and 1 μM **1c** in the presence of α-MSH and IBMX for 48 h, and analyzed for melanin content and cellular tyrosinase activity. In this experiments, α-MSH and IBMX treatment notably increased melanin content, while **1c** treatment noticeably attenuated melanin content in B16F10 melanoma cells in a dose-dependent (Figure 5A). The melanin contents were 171.30%, after α-MSH and IBMX treatment, and reduced to 118.19%, 107.29%, 98.15%, and 88.09% after treatment with 10 μM kojic acid or 0.04 μM, 0.2 μM, and 1 μM **1c**, respectively. According to these results, **1c** significantly inhibited melanin biosynthesis. Melanin overproduction was found to stimulate the cellular tyrosinase [35]. For this reason, reduction of tyrosinase activity is an efficient strategy in development of anti-melanogenic agents [36,37]. l-Tyrosine and l-DOPA are sequentially generated substrates that positively regulate melanogenesis and modulates melanocyte function through overlapping substrates [38]. To study this induction, we designed l-DOPA oxidation protocols to examine the inhibitory activity of **1c** against tyrosinase in α-MSH and IBMX-induced B16F10 melanoma cells. After 48 h of **1c** treatment, we measured the intracellular tyrosinase activity. As demonstrated in Figure 5B, after 48 h of treatment with **1c** (0.04, 0.2, and 1 μM) and α-MSH and IBMX, the intracellular tyrosinase activity decreased dose-dependently comparison to control. The levels of intracellular tyrosinase activity were 310.39%, after α-MSH and IBMX treatment, and were reduced to 225.54%, 225.78%, 157.79%, and 132.71% after treatment with 10 μM kojic acid or 0.04 μM, 0.2 μM, and 1 μM **1c**, respectively. These results indicated that cellular tyrosinase inhibition by **1c** was responsible for its anti-melanogenesis potential. In this study, we used B16F10 cells instead of human normal skin cells (melanocyte), which produce the melanin, because these cells have several common aspects of normal melanocytes [30,39,40,41]. To the best of our knowledge, this study is the first report on anti-melanogenesis activity of thiophene chalcone derivatives by inhibiting tyrosinase in the cell base experiment system.

## 3. Material and Methods

### 3.1. Chemicals and Reagents

Mushroom tyrosinase (EC 1.14.18.1), α-melanocyte stimulating hormone (α-MSH), 3-isobutyl-1-methylxanthine (IBMX), l-tyrosine, l-3,4-dihydroxyphenylalanine (l-DOPA), and kojic acid were purchased from Sigma-Aldrich Co. (St. Louis, MO, USA). Dulbecco’s modified Eagle’s medium (DMEM), fetal bovine serum (FBS), and streptomycin were obtained from WELGENE Inc. (Gyeongsan-si, Korea). K_2_HPO_4_ was obtained from Junsei Chemical Co. Ltd. (Tokyo, Japan), and KH_2_PO was acquired from Yakuri Pure Chemicals Co. Ltd. (Osaka, Japan). All chemistry reactions were performed under a nitrogen atmosphere and checked by thin-layer chromatography (TLC) accomplished on Merck precoated 60F_254_ plates (20 × 20 cm, 0.25 mm) (Merck, Darmstadt, Germany).

### 3.2. Chemistry

All the reagents were achieved commercially and used without further purification. High-resolution MS (HRMS) data were obtained on a 6530 Accurate Mass quadrupole time-of-flight liquid-chromatograph mass spectrometer (Agilent Technologies, Santa Clara, CA, USA). ^1^H and ^13^C nuclear magnetic resonance (NMR) spectral were recorded in CD_3_OD (*δ*_H_ 3.30 and *δ*_C_ 49.0), CDCl_3_ (*δ*_H_ 7.24 and *δ*_C_ 77.0), and DMSO-*d*_6_ (*δ*_H_ 2.50 and *δ*_C_ 39.7) on a Varian Unity INOVA 400 spectrometer or a Varian Unity AS500 spectrometer (Agilent Technologies).

#### 3.2.1. Synthetic Procedure for Compounds **1a**, **1b**, and **1d**

Method A: A suspension of substituted benzaldehyde, **a**, **b**, or **d** (0.66~0.82 mmol) and 2-acetylthiophene (1.0 equiv.) in 1.0 M HCl acetic acid solution (1.0 mL/1.0 mmol of benzaldehyde) was stirred at room temperature (**a**) or 60 °C (**b** and **d**) for 27–96 h. Water was added to the reaction mixture, neutralized with a NaHCO_3_ aqueous solution and extracted with methylene chloride. The organic layer was dehydrated over MgSO_4_, filtered, and dried in vacuo. The residue was recrystallized to obtain compounds **1a**, **1b** and **1d** in yields of 5~88%.

#### 3.2.2. Synthetic Procedure for Compounds **1e**–**h**

Method B: A 1.0 M NaOH aqueous solution (1.6 mL) was added to a suspension of substituted benzaldehyde, **e**, **f**, **g** or **h** (0.80 mmol) and 2-acetylthiophene (0.80 mmol) in methanol (2.0 mL). After the reaction mixture was stirred at room temperature for 8 h (**e**), or 60 °C for 1–6 h (**f**–**h**), water was added to the reaction mixture and neutralized with 2.0 M HCl. The precipitates were filtered and eluted with hexane and methylene chloride or refined by silica gel column chromatography using hexane and ethyl acetate to provide compounds **1e**–**h** with yields of 14~32%.

#### 3.2.3. Synthetic Procedure for (*E*)-3-(2,4-Dihydroxyphenyl)-1-(thiophen-2-yl)prop-2-en-1-one (**1c**)

To a stirred solution of 2-acetylthiophene (105 mg, 0.83 mmol) in MeOH (2.0 mL) were added to 20% NaOH aqueous solution (1.25 mL) and 2-hydroxy-4-((tetrahydro-2*H*-pyran-2-yl)oxy)benzaldehyde (**c**′, 203 mg, 0.91 mmol), successively. After being stirred at room temperature for 48 h, the reaction mixture was divided between methylene chloride and water. The organic fraction was dried out anhydrous MgSO_4_, filtered, and evaporated under reduced pressure to give a crude product **1c**′, which was used for the next reaction without further refinement. A total of 2.0 M HCl aqueous solution (1.0 mL) and 1,4-dioxane (1.0 mL) were added to the crude product **1c**′. After being stirred at room temperature for 30 min, the reaction mixture was partitioned between methylene chloride and water. The organic layer was desiccated over anhydrous MgSO_4_, filtered, and evaporated under condensed pressure. The resultant residue was separated by silica gel column chromatography with hexane and ethyl acetate (1:1) as eluent, in collection of **1c** (57.2 mg, 45%) as a brown solid.

*(E)-3-(4-Hydroxyphenyl)-1-(thiophen-2-yl)prop-2-en-1-one* (**1a**). Yellow solid; reaction time, 27 h; yield, 88%; melting point, 177.2–180.0 °C; ^1^H-NMR (400 MHz, CD_3_OD) *δ* 8.03 (dd, 1 H, *J* = 4.0, 1.2 Hz, 5′′-H), 7.82 (dd, 1 H, *J* = 4.8, 1.2 Hz, 3′′-H), 7.72 (d, 1 H, *J* = 15.6 Hz, 3-H), 7.59 (d, 2 H, *J* = 8.4 Hz, 2′-H, 6′-H), 7.48 (d, 1 H, *J* = 15.6 Hz, 2-H), 7.21 (dd, 1 H, *J* = 4.8, 4.0 Hz, 4′′-H), 6.82 (d, 2 H, *J* = 8.4 Hz, 3′-H, 5′-H); ^13^C-NMR (100 MHz, CD_3_OD) *δ* 183.2, 160.6, 145.8, 144.7, 134.3, 132.6, 130.7, 128.4, 126.3, 118.0, 115.8; HR-MS (ESI+) *m*/*z* C_13_H_11_O_2_S (M + H)^+^ calcd 231.0474, obsd 231.0471.

*(E)-3-(3,4-Dihydroxyphenyl)-1-(thiophen-2-yl)prop-2-en-1-one* (**1b**). Khaki-colored solid; reaction time, 96 h; yield, 18%; melting point, 182.5–185.7 °C; ^1^H-NMR (400 MHz, DMSO-*d*_6_) *δ* 9.17 (s, 1 H, OH), 9.09 (s, 1 H, OH), 8.21 (d, 1 H, *J* = 4.0 Hz, 5′′-H), 7.97 (d, 1 H, *J* = 4.8 Hz, 3′′-H), 7.56 (d, 1 H, *J* = 16.0 Hz, 3-H), 7.52 (d, 1 H, *J* = 16.0 Hz, 2-H), 7.26–7.23 (m, 2 H, 2′-H, 4′′-H), 7.15 (d, 1 H, *J* = 8.4 Hz, 6′-H), 6.78 (d, 1 H, *J* = 8.4 Hz, 5′-H); ^13^C-NMR (100 MHz, DMSO-*d*_6_) *δ* 182.1, 149.5, 146.6, 146.3, 144.8, 135.5, 133.6, 129.5, 126.8, 122.9, 118.9, 116.4, 116.3; HR-MS (ESI+) *m*/*z* C_13_H_11_O_3_S (M + H)^+^ calcd 247.0423, obsd 247.0419.

(*E)-3-(2,4-Dihydroxyphenyl)-1-(thiophen-2-yl)prop-2-en-1-one* (**1c**). Brown solid; reaction time, 48 h for 1st step (condensation with acetylthiophene), 30 min for 2nd step (deprotection of THP); two-step yield, 27.9% (1st step: 62%, 2nd step: 45%); melting point, 140.5–142.7 °C; ^1^H-NMR (400 MHz, DMSO-*d*_6_) *δ* 10.18 (s, 1 H, OH), 9.95 (s, 1 H, OH), 8.09 (d, 1 H, *J* = 4.0 Hz, 5′′-H), 7.93 (d, 1 H, *J* = 4.8 Hz, 3′′-H), 7.93 (d, 1 H, *J* = 15.6 Hz, 3-H), 7.66 (d, 1 H, *J* = 8.4 Hz, 6′-H), 7.54 (d, 1 H, *J* = 15.6 Hz, 2-H), 7.23 (dd, 1 H, *J* = 4.8, 4.0 Hz, 4′′-H), 6.35 (d, 1 H, *J* = 2.0 Hz, 3′-H), 6.28 (dd, 1 H, *J* = 8.4, 2.0 Hz, 5′-H); ^13^C-NMR (100 MHz, DMSO-*d*_6_) *δ* 182.3, 162.2, 159.9, 146.9, 139.8, 135.0, 132.9, 130.9, 129.4, 117.4, 113.8, 108.7, 103.1; HR-MS (ESI+) *m*/*z* C_13_H_11_O_3_S (M + H)^+^ calcd 247.0423, obsd 247.0419.

*(E)-3-(4-Hydroxy-3-methoxyphenyl)-1-(thiophen-2-yl)prop-2-en-1-one* (**1d**). Greenish yellow solid; reaction time, 96 h; yield, 5%; melting point, 132.8–135.2 °C; ^1^H-NMR (400 MHz, DMSO-*d*_6_) *δ* 9.68 (s, 1 H, OH), 8.26 (d, 1 H, *J* = 4.0 Hz, 5′′-H), 7.98 (d, 1 H, *J* = 4.8 Hz, 3′′-H), 7.66 (d, 1 H, *J* = 15.6 Hz, 3-H), 7.61 (d, 1 H, *J* = 15.6 Hz, 2-H), 7.46 (s, 1 H, 2′-H), 7.28–7.24 (m, 2 H, 6′-H, 4′′-H), 6.80 (d, 1 H, *J* = 8.0 Hz, 5′-H), 3.83 (s, 3 H, OCH_3_); ^13^C-NMR (100 MHz, DMSO-*d*_6_) *δ* 182.2, 150.5, 148.7, 146.6, 144.7, 135.7, 133.7, 129.4, 126.7, 124.8, 119.1, 116.3, 112.5, 56.5; HR-MS (ESI+) *m*/*z* C_14_H_13_O_3_S (M + H)^+^ calcd 261.0580, obsd 261.0580.

*(E)-3-(3-Hydroxy-4-methoxyphenyl)-1-(thiophen-2-yl)prop-2-en-1-one* (**1e**). Light yellow solid; reaction time, 8 h; yield, 14%; melting point, 153.5–158.2 °C; ^1^H-NMR (400 MHz, DMSO-*d*_6_) *δ* 9.13 (s, 1 H, OH), 8.25 (d, 1 H, *J* = 3.6 Hz, 5′′-H), 7.98 (d, 1 H, *J* = 4.4 Hz, 3′′-H), 7.61 (d, 1 H, *J* = 16.0 Hz, 3-H), 7.57 (d, 1 H, *J* = 16.0 Hz, 2-H), 7.30 (s, 1 H, 2′-H), 7.27–7.24 (m, 2 H, 6′-H, 4′′-H), 6.96 (d, 1 H, *J* = 8.0 Hz, 5′-H), 3.80 (s, 3 H, OCH_3_); ^13^C-NMR (100 MHz, DMSO-*d*_6_) *δ* 182.2, 151.0, 147.3, 146.5, 144.3, 135.7, 133.8, 129.5, 128.1, 122.9, 119.9, 115.6, 112.5, 56.4; HR-MS (ESI+) *m*/*z* C_14_H_13_O_3_S (M + H)^+^ calcd 261.0580, obsd 261.0574.

*(E)-3-(4-Hydroxy-3,5-dimethoxyphenyl)-1-(thiophen-2-yl)prop-2-en-1-one* (**1f**). Beige-colored solid; reaction time, 6 h; yield, 27%; melting point, 143.6–147.3 °C; ^1^H-NMR (500 MHz, CDCl_3_) *δ* 7.83 (d, 1 H, *J* = 4.0 Hz, 5′′-H), 7.69 (d, 1 H, *J* = 15.5 Hz, 3-H), 7.60 (d, 1 H, *J* = 5.0 Hz, 3′′-H), 7.24 (d, 1 H, *J* = 15.5 Hz, 2-H), 7.09 (dd, 1 H, *J* = 5.0. 4.0 Hz, 4′′-H), 6.81 (s, 2 H, 2′-H, 6′-H), 6.14 (s, 1 H, OH), 3.87 (s, 6 H, 2 × OCH_3_); ^13^C-NMR (100 MHz, CDCl_3_) *δ* 182.2, 147.5, 145.9, 144.9, 137.8, 133.9, 131.8, 128.4, 126.4, 119.7, 105.8, 56.6; HR-MS (ESI+) *m*/*z* C_15_H_15_O_4_S (M + H)^+^ calcd 291.0686, obsd 291.0680.

*(E)-3-(3-Bromo-4-hydroxyphenyl)-1-(thiophen-2-yl)prop-2-en-1-one* (**1g**). Light yellow solid; reaction time, 1 h; yield, 23%; melting point, 195.2–199.6 °C; ^1^H-NMR (500 MHz, DMSO-*d*_6_) *δ* 10.91 (s, 1 H, OH), 8.32 (d, 1 H, *J* = 4.0 Hz, 5′′-H), 8.15 (s, 1 H, 2′-H), 8.02 (d, 1 H, *J* = 5.0 Hz, 3′′H), 7.74 (d, 1 H, *J* = 15.5 Hz, 3-H), 7.67 (d, 1 H, *J* = 8.5 Hz, 6′-H), 7.61 (d, 1 H, *J* = 15.5 Hz, 2-H), 7.29 (dd, 1 H, *J* = 5.0, 4.0 Hz, 4′′-H), 6.99 (d, 1 H, *J* = 8.5 Hz, 5′-H); ^13^C-NMR (100 MHz, DMSO-*d*_6_) *δ* 182.1, 157.1, 146.4, 142.7, 135.9, 134.1, 133.8, 131.1, 129.5, 128.0, 120.5, 117.1, 110.8; HR-MS (ESI+) *m*/*z* C_13_H_10_BrO_2_S (M + H)^+^ calcd 308.9579, obsd 308.9574, (M + 2 + H)^+^ calcd 310.9559, obsd 310.9551.

*(E)-3-(3,5-Dibromo-4-hydroxyphenyl)-1-(thiophen-2-yl)prop-2-en-1-one* (**1h**). Yellow solid; reaction time, 3 h; yield, 32%; melting point, 269.3–272.0 °C; ^1^H-NMR (400 MHz, DMSO-*d*_6_) *δ* 10.49 (brs, 1 H, OH), 8.34 (d, 1 H, *J* = 4.0 Hz, 5′′-H), 8.13 (s, 2 H, 2′-H, 6′-H), 8.01 (d, 1 H, *J* = 4.8 Hz, 3′′-H), 7.81 (d, 1 H, *J* = 15.6 Hz, 3-H), 7.56 (d, 1 H, *J* = 15.6 Hz, 2-H), 7.28 (dd, 1 H, *J* = 4.8, 4.0 Hz, 4′′-H); ^13^C-NMR (100 MHz, DMSO-*d*_6_) *δ* 182.1, 153.4, 146.3, 141.2, 136.3, 134.5, 133.5, 129.8, 129.5, 122.1, 112.8; HR-MS (ESI+) *m*/*z* C_13_H_9_Br_2_O_2_S (M + H)^+^ calcd 386.8685, obsd 386.8677, (M + 2 + H)^+^ calcd 388.8664, obsd 388.8648, (M + 4 + H)^+^ calcd 390.8644, obsd 390.8625.

### 3.3. Biological Assessment

#### 3.3.1. Mushroom Tyrosinase Inhibitory Assay

We investigated the effects of (*E*)-3-phenylthiophenylprop-2-en-1-one derivatives (**1a**–**h**) on oxidization activity of tyrosinase using l-tyrosine or l-DOPA as substrates as described in our aforementioned study with a modified method [20,21]. Concisely, 10 μL of a detailed concentration of each compound (0.0005–50 μM) and 20 μL of mushroom tyrosinase (1000 Units/mL) in 50 mM phosphate buffer (pH 6.5) were added to 170 μL of an assay mixture buffer in a 96-well plate. The ratio of 1mM l-tyrosine or l-DOPA solution, 50 mM potassium phosphate buffer (pH 6.5), and purified water was 10:10:9. The reaction mixtures were keep warm at 37 °C for 20 min. The absorbance of the mixture at 492 nm was measured using a microplate reader. The tyrosinase inhibitory activity (%) was calculated as (1 − Abs_sample_/Abs_control_) × 100%. The degree of inhibition of the sample was stated as the concentration required for 50% inhibition (IC_50_).

#### 3.3.2. Enzyme Kinetic Analysis in Mushroom Tyrosinase Inhibition

In order to estimate the kinetic mechanism, two kinetic methods using Lineweaver-Burk and Dixon plots were used [21,32]. Using Lineweaver-Burk double reciprocal plots [a plot of 1/enzyme velocity (1/*V*) vs. 1/substrate concentration (1/[*S*])], the type of inhibition was determined using various concentrations of l-tyrosine (0.5, 1 and 2 mM) and l-DOPA (0.5, 1, and 2 mM), as substrates, in the absence and presence of various concentrations of **1c** (3.2–80 nM; for l-tyrosine, 0.4–10 μM; for l-DOPA). The Dixon plot for inhibition of tyrosinase was obtained using various concentrations of l-tyrosine (0.5, 1 and 2 mM) and l-DOPA (0.5, 1 and 2 mM). The concentrations of **1c** were as follows: 3.2–80 nM; for l-tyrosine, 0.4–10 μM; for l-DOPA. The enzymatic procedures comprised of the formerly described tyrosinase test methods. The inhibition constant (*K*_i_) was resolute using Dixon plots.

#### 3.3.3. In Silico Molecular Docking Simulation of Tyrosinase Inhibition

To determine the possible molecular mechanism and the active groups of **1c** responsible for inhibition of tyrosinase, molecular docking simulations were accomplished using AutoDock 4.2 program (AutoDock4 and AutoDockTools4, OpenEye Scientific Software, SantaFe, NM, USA). X-ray crystallographic structures show quaternary ligand binding to aromatic residues in the active-site gorge of tyrosinase (PDB ID: 2Y9X) as determined using the RCSB Protein Data Bank (http://www.rcsb.org/adb) [42]. Docking simulations were performed between tyrosinase and kojic acid or **1c**. For the docking procedure: (1) the 2D structure of **1c** was converted into a 3D structure, (2) charges were calculated, and (3) hydrogen atoms were added using ChemOffice software (http://www.cambridgesoft.com). LigandScout 4.1.5 was used to forecast possible interactions between ligands and tyrosinase and to identify pharmacophores.

#### 3.3.4. Cell Culture

Murine melanoma B16F10 cells were purchased from the Korean Cell Line Bank (Seoul, Korea). The cells were cultured in DMEM containing 10% FBS and 1% streptomycin, and then incubated at 37 °C with 5% CO_2_. The melanoma cells were plated at 95% confluency for all the experiments.

#### 3.3.5. Assay for Cell Viability

Cell viability was assessed with the EZ-Cytox assay kit (Daeillab Service, Seoul, Korea) as previously described [21]. In brief, B16F10 cells were seeded in 96-well plates (Corning, NY, USA) at a density of 1 × 10^4^ cells/well for 24 h. The cells were fed fresh, serum-free DMEM that contained different concentrations (up to 100 μM) of **1c**, and incubated for 24 h or 48 h. Then, 10 μL EZ-Cytox solution was added to each well and the cells were incubated for 2–4 h. The absorbance of cells in the absence of treatment was regarded as 100% cell survival. Each treatment was achieved in triplicate and each experiment was repeated three times. The 50% cytotoxic concentration (CC_50_) was defined as the test compound concentration required for the reduction of cell viability by half. Thus, CC_50_ value of **1c** were calculated by regression analysis.

#### 3.3.6. Inhibitory Activity of Melanin Contents in B16F10 Melanoma Cells

Cellular melanin content was determined as described previously [43]. Cells were seeded in six-well culture plates (Nunc, Denmark) at 2 × 10^5^ cells/well for 24 h. To determine the inhibitory effect of **1c** on melanogenesis, fresh medium was replaced with medium containing **1c** (0.04, 0.2, and 1 μM) or kojic acid (10 μM) as a positive control for 1 h, and then stimulated with α-MSH (5 μM) and IBMX (200 μM) for 48 h. Cells were washed twice with PBS. After centrifugation, cell pellets were disrupted in 100 μL of 1N NaOH, and incubated at 60 °C for 1 h and mixed to solubilize the melanin. Absorbance at 405 nm was compared with a standard curve for synthetic melanin (TECAN, Salzburg, Austria).

#### 3.3.7. Inhibitory Activity of Cellular Tyrosinase Activity Assay in B16F10 Melanoma Cells

Intracellular tyrosinase inhibitory activity assay was achieved by measuring the rate of oxidation of l-DOPA [44]. Cells at a density of 1 × 10^5^ cells/well were plated in 6-well plates (Nunc, Denmark) and incubated overnight. Then, cells were treated with various concentrations of **1c** (0.04, 0.2, and 1 µM) or kojic acid (10 µM) for 1 h, then stimulated with α-MSH (5 µM) and IBMX (200 µM) for 48 h. The cells were washed with PBS and lysed in a solution containing 100 µL of 50 mM phosphate buffer (pH 6.5), 0.1 mM phenylmethylsulfonylfluoride (PMSF), and 1% Triton X-100. The cells lysates were employed in a deep freezer (−80 °C) for 1 h. After defrosting the cell lysates, the cellular extracts were purified by centrifugation at 12,000 rpm for 30 min at 4 °C. A total of 80 µL of supernatant and 20 µL of l-DOPA (2 mg/mL) were added to a 96-well plate, and the absorbance at a wavelength of 492 nm was measured every 10 min during 1 h using an ELISA plate reader (TECAN, Salzburg, Austria). Total protein content was determined using BCA protein assay reagent (Thermo Scientific, Rockford, IL, USA).

#### 3.3.8. Statistical Analysis

All statistical analyses were presented as mean ± S.E.M of at least three separate experiments. The data were analyzed by one-way analysis of variance (ANOVA) for the differences between treatments followed by the Bonferroni post-hoc test (SPSS, Chicago, IL, USA). A value of *p* < 0.05 indicated considered to indicated a statistically significance.

## 4. Conclusions

In conclusion, this study first reports the hyperpigmentary mechanism of thiophene chalcone derivatives in B16F10 melanoma cells. Among these derivatives (**1a**–**h**), **1c** exerted potent tyrosinase inhibitory effect with IC_50_ values (0.013 μM for tyrosine hydroxylase and 0.93 μM for dopa oxidase) significant lower than values achieved for the reference inhibitor kojic acid. **1c** was shown as a competitive inhibitor against tyrosinase and fit well into the active sites of tyrosinase according to enzyme kinetic studies and molecular docking results. In addition, **1c** inhibited cellular tyrosinase activity. Collectively, **1c** could be useful as a tyrosinase inhibiting agent for the purpose of prevention and treatment of skin pigmentation and inhibition of melanogenesis. Further in vivo research and clinical trials should be carried out to clarify the efficacy, safety, and precise molecular mechanism of anti-melanogenesis effects of **1c**.

## Figures and Tables

**Table 1:**
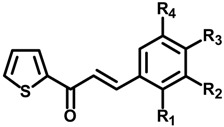
Substitution pattern of the substituted 3-phenyl-1-thiophen-2-yl-propenone derivatives (**1a**–**h**).

Compounds	R_1_	R_2_	R_3_	R_4_	IC_50_ (μM) ^a^	IC_50_ (μM) ^b^
**1a**	H	H	OH	H	46.16 ± 0.55	60.05 ± 7.85
**1b**	H	OH	OH	H	75.72 ± 2.46	103.44 ± 8.47
**1c**	OH	H	OH	H	0.013 ± 0.64	0.93 ± 0.22
**1d**	H	OMe	OH	H	98.78 ± 2.11	>200
**1e**	H	OH	OMe	H	17.44 ± 1.81	28.72 ± 1.98
**1f**	H	OMe	OH	OMe	77.91 ± 8.74	>200
**1g**	H	Br	OH	H	>200	112.09 ± 14.27
**1h**	H	Br	OH	Br	>200	>200
Kojic acid ^c^					22.84 ± 0.09	24.57 ± 0.23

The IC_50_ values (μM) were calculated from a log dose inhibition curve using l-tyrosine ^a^ and l-DOPA ^b^ as a substrate, respectively, and are as means ± standard error of the mean (SEM) of triplicate experiments. ^c^ Used as positive control.

**Table 2:** Enzyme kinetic analysis of **1c** against mushroom tyrosinase.

	Inhibition Type ^a^ (*K*_i_) ^b^	
	l-Tyrosine	l-DOPA
**1c**	Competitive (5.01 nM)	Competitive (1.76 μM)
Kojic acid ^c^	NT ^d^	NT ^d^

^a^ Determined by the Lineweaver-Burk plot. ^b^ Determined by the Dixon plot. ^c^ Kojic acid was used as a positive control. ^d^ Not tested.

**Table 3:** Tyrosinase inhibitory activity of **1c**.

	Binding Energy ^a^ (kcal/mol)	No. of H-Bond ^b^	H-Bond Interacting ^b^ Residues	Van der Waals Bond Interaction Residues ^b^
**1c**	−5.9	2	ASN260, MET280	MET257, VAL248, PHE264, VAL283, ALA286
Kojic acid ^c^	−4.2	2	MET280	HIS263

^a^ Binding energy indicate binding affinity and capacity for the active site of tyrosinase enzyme. ^b^ The number of hydrogen bonds, and all amino acid residues from the enzyme-inhibitor complex were determined by using AutoDock 4.2 program ^c^ Reported competitive type inhibitor.
